# Community case study: Our Wave, an online platform to facilitate survivor disclosure of sexual assault and trauma recovery

**DOI:** 10.3389/fpubh.2024.1458510

**Published:** 2024-12-18

**Authors:** Laura Sinko, Olga V. Naidenko, Brendan Michaelsen, Cassandra Marzke, Madeline Hart, Brooke Tweedie, Kyle Linton

**Affiliations:** ^1^Our Wave, Raleigh, NC, United States; ^2^Department of Nursing, Temple University College of Public Health, Philadelphia, PA, United States

**Keywords:** sexual assault disclosure, SV, sexual harm, online community, trauma recovery, healing

## Abstract

This community case study introduces Our Wave, an online platform that provides a safe, anonymous space for survivors of sexual harm to share their stories, reflect on their healing journeys, and connect with others. Designed to empower survivors, the platform allows users to post anonymous stories or visual media, ask questions, and send messages of hope, all while prioritizing privacy and security. It also aims to create a broader impact by analyzing shared narratives to detect patterns, identify best practices for healing, and inform global approaches to SV recovery. With over 100,000 members and over 1,200 stories and forms of media shared, the platform has become a valuable resource for survivors. Using mixed-methods analysis of platform usage data and anonymous user feedback surveys (*n* = 288), we examined patterns of engagement and impact. Analysis revealed that 57% of story submissions included “Messages of Healing” and 52% included “Messages of Hope,” with survey responses indicating that anonymity and peer support were crucial factors in facilitating disclosure. Research shows that the social and emotional dynamics of SV disclosure, as well as the responses from others, play a crucial role in a survivor’s healing. By focusing on confidentiality, trauma-informed support, and community-building, Our Wave offers a safe space for survivors to heal, connect, and access resources to support their journey toward recovery.

## Introduction

Sexual violence (SV) represents a pervasive public health crisis affecting individuals across demographic groups worldwide. Current epidemiological data indicates that approximately one in three women, one in six men, and one in two gender-diverse individuals experience SV during their lifetime (1, 21). Research demonstrates that survivors of SV seek to tell their stories not just for individual healing but also to challenge systems that perpetuate violence and create broader social change (2). Storytelling can allow survivors to reclaim power and control over their narrative after experiencing violence that is inherently disempowering.

The health consequences of SV are well-documented, encompassing both immediate and long-term impacts on survivors’ physical, mental, and social wellbeing (3, 22). Research demonstrates that survivors of SV face multiple barriers to accessing support services, including fear of retaliation or negative social responses, concerns about confidentiality and privacy, structural barriers such as geographic isolation or limited service availability, and pervasive stigma and shame associated with disclosure(23–26).

The process of disclosing SV experiences plays a crucial role in survivors’ recovery trajectories. Studies indicate that the initial disclosure experience and subsequent social responses significantly influence psychological outcomes, help-seeking behaviors, and overall healing (4, 5). Research shows that negative social reactions to SV disclosure can have profound negative effects on recovery and play a crucial role in the development of PTSD symptoms (6, 7). Moreover, negative responses to disclosure can cause healing disengagement, decreasing future help-seeking while increasing risks of maladaptive coping strategies (8). These risks extend to online spaces, where survivors may face hostile responses or “trolling” that can retraumatize them (9).

Recent research examining online communities has found that anonymous digital spaces provide survivors with three key benefits: finding supportive community, seeking advice from peers, and having a platform for storytelling (10). These online spaces allow survivors to disclose their experiences when they may not be able to do so elsewhere, with many expressing gratitude for having their stories heard and validated by others.

The digital age has transformed how survivors engage with support services and share their experiences. Online platforms have emerged as crucial spaces for disclosure, particularly following social movements like #MeToo (11, 12). For example, in the 24 h after the story broke about Harvey Weinstein’s history of sexual abuse, #MeToo was used over 500,000 times on Twitter and over 12 million times on Facebook (13). However, not all survivors who want to share their stories online can do so, often due to fears of public exposure, linking SV to their online identities, retaliation, or negative responses they may receive (14).

Through the lens of the Theory of Planned Behavior (15), survivor storytelling in online spaces may facilitate both individual and collective empowerment by: changing attitudes about breaking silence, challenging stigmatizing social norms through shared experiences, and increasing survivors’ perceived control over their stories and healing journeys. Research indicates that online disclosure offers unique advantages for survivors, including enhanced control over narrative and timing, reduced immediate social pressure, access to broader support networks, and opportunities for anonymous disclosure (12, 14, 16, 17). However, existing online platforms present significant challenges, including limited privacy protections, insufficient content moderation, lack of trauma-informed design, and inadequate integration with professional support services (24, 25).

Our Wave, established in 2019, addresses these gaps through a specialized online platform designed specifically for survivors of SV. The platform integrates evidence-based approaches to trauma support with innovative digital tools, prioritizing user safety, anonymity, and empowerment. Through a trauma-informed, survivor-centered approach, Our Wave aims to facilitate safe, anonymous disclosure of SV experiences, foster connection and community among survivors, provide accessible education and resources, generate insights into patterns of violence and pathways to healing, and support evidence-based research while protecting survivor privacy. This case study examines Our Wave’s implementation, impact, and implications for online survivor support services.

## Context

The mission of Our Wave is to be a safe harbor for survivors of sexual harm by fostering connection: with the self through reflection, with others through building community, and with the world by connecting the dots between stories to collaborate on best practices for healing, detect patterns, and make a global impact. The Our Wave platform allows victims to safely share stories and/or visual media, ask anonymous questions, reflect on their healing journey, and send messages of hope to others. All submissions are reviewed by trained Our Wave team members, who remove identifying information and provide content warnings when applicable. Other platform features include grounding exercises before reading or sharing stories, escape buttons to leave the site quickly in case of an emergency, story tags to link communities together, online exhibits to educate the public about survivorship, and resource lists connecting victims to local and national organizations.

Unlike typical social media platforms including Reddit, Instagram, TikTok, and Twitter, Our Wave’s story sharing and data tagging functionalities were created specifically for survivors of sexual harm vs. a broader general audience. There are other grass-roots technical solutions (WordPress plugins, Google Forms, etc.) that are utilized by advocacy organizations to facilitate disclosure but they often do not have custom functionality catered to support survivor needs.

Since Our Wave platform launched in 2019, the website has gathered over 1,200 survivor stories, has answered over 280 anonymous questions about survivorship, and has had more than 100 thousand community members visit. See a demographic breakdown of story submitters in Table 1. Survivors also can opt in to allow the Our Wave team to use their stories for research, to better understand patterns of perpetration, gaps in care, and healing needs. Currently, over 1,100 of these stories have permission to be used for this purpose. This significant dataset enables researchers to better understand patterns of perpetration, identify gaps in care, and assess healing needs, providing valuable insights that are often scarce due to underreporting of SV. By contributing to this research, survivors not only share their experiences but also play a crucial role in shaping more effective prevention strategies, support services, and policy initiatives, ultimately leading to systemic improvements in addressing SV.

**Table 1:** Breakdown of current story submitters demographics.

Demographic	Frequency
Age range	
Child (under 12)	323
Teenager (13–17)	265
Young adult (18–29)	291
Adult (30–60)	209
Older adult (60+)	10
Race	
Asian	69
Arab/Middle Eastern/North African	4
Black/African/Caribbean	85
Hispanic/Latina/Spanish	88
American Indian/Alaskan Native	18
Native Hawaiian/Other Pacific Islander	2
Two or more races	61
White	526
Sexual orientation	
LGBTQ+	92
Straight	477
Lesbian/Gay	44
Bisexual	189
Pansexual	46
Queer	50
Asexual	14
Gender	
Man	78
Woman	728
Transgender	28
Non-binary	56
Gender-fluid	12
Genderqueer	11
Other forms of self-identification	
A Person with a physical disability	24
A Person who is neurodivergent	70
A Person who is deaf/hard of hearing	2
A Person who is blind or has a visual impairment	6
A Person with a speech or language impairment	4
A Person with an intellectual or developmental disability	16
An Immigrant	6

## Key programmatic elements

### Anonymous story sharing

Survivors share whatever details they want about their story through narrative or visual media as well as what healing means to them. As part of the sharing process, survivors can provide additional context by self-selecting tags including their demographic information (e.g., race, ethnicity, sexual orientation, age range) and details relating the assault or abuse that has taken place, such as relationship to the perpetrator, and general location, such as whether the violent event took place at home, in school or another location. Survivors can also opt in to allow their data to be used for research, to be able to better understand patterns and trends relating to survivorship, violence perpetration, and healing. The Our Wave team securely redacts any story identifying information, provides tailored resources to the story submitter, and posts it on the Our Wave site, where survivors can read others’ stories, provide support through reacting to stories, and filter based on the survivor-provided tags. Survivors can periodically post updates to their stories, to demonstrate longitudinally how survivors’ needs and experiences may change over time. See potential story tags in Table 2.

**Table 2:** Story tags survivors can select.

Tag type	Response options (select all that apply)
Where sexual harm experience occurred	Home At Someone Else’s Home At Work At School/University In a Bar/Restaurant In the Military At a Social Event Traveling In a Service Setting Other
Relationship to the person who harmed them	Stranger Acquaintance Non-Romantic Friend Casual/First Date Romantic Partner Family Member Authority Figure Colleague Minor
Violence that co-occurred with the sexual harm experience	Physical harm Emotional abuse Financial abuse Human trafficking
Developmental stage of the submitter when the harm occurred	A Child A Teenager A Young Adult An Adult A Senior
Story submitter race/ethnicity	Asian Black/African Hispanic/Latino/Spanish American Indian/Alaska Native Two or More Races Native Hawaiian/Other Pacific Islander White
Story submitter sexual orientation	LGBTQ+ Straight Lesbian/Gay Bisexual Pansexual Queer
Story submitter gender	A Man A Woman Transgender Non-binary Gender-fluid
Other identity-related story submitter tag options	A Person with a disability A Person who is neurodivergent An Immigrant

### Survivor Q & A

**Figure 1:**
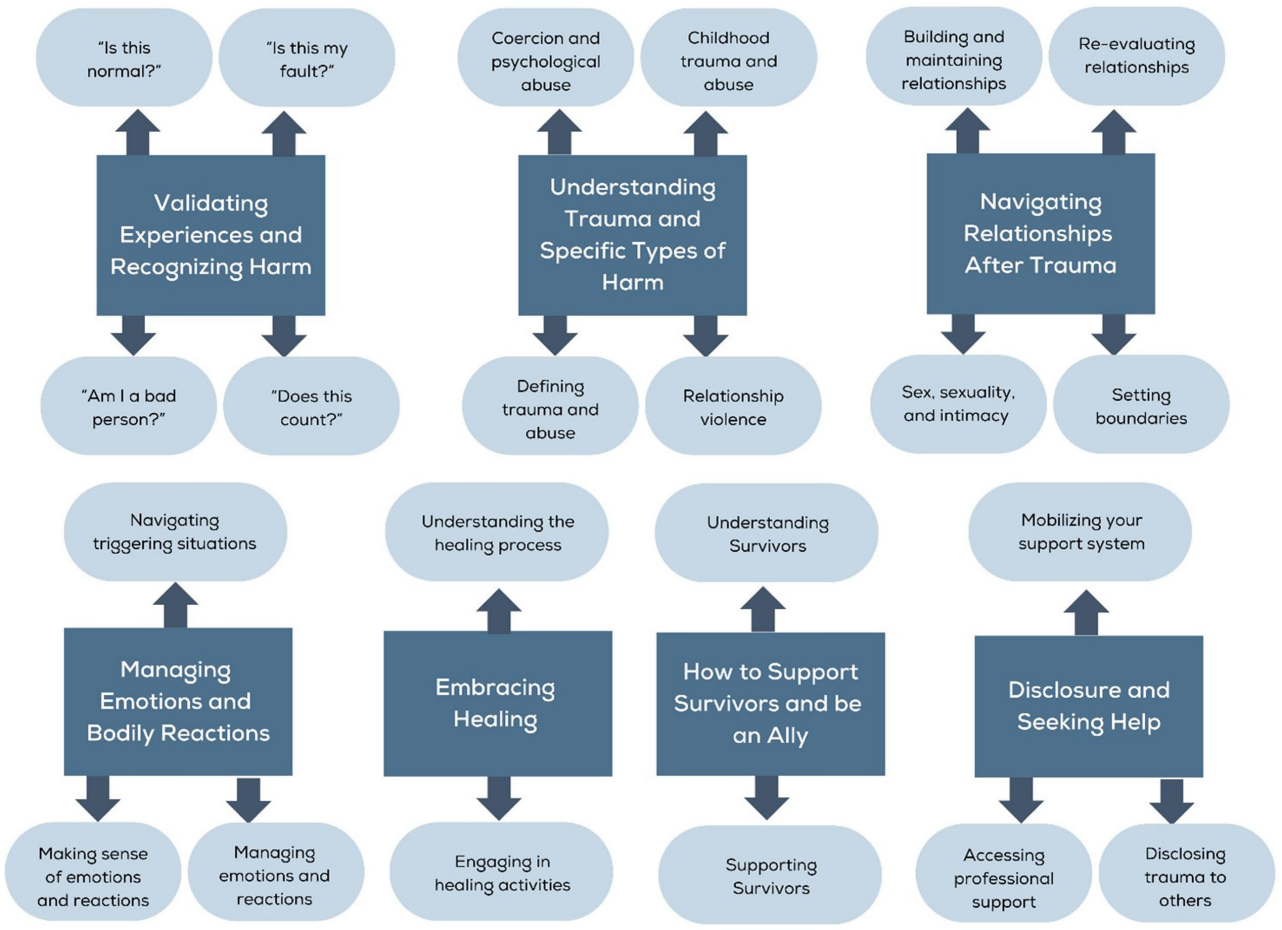
Themes across submitted questions.

The website also features an anonymous survivor question and answer section, where survivors can ask questions of mental health and trauma experts relating to their survivorship experiences, understanding trauma and its consequences, making meaning, seeking help and healing, and supporting other survivors. These questions are reviewed by the Our Wave team and evidence-informed responses are posted 3–5 days later. We conducted a content analysis of our first 100 questions to understand core themes across questions (see Figure 1). Question domains found included questions relating to: (1) validating personal traumatic experiences and recognizing harm that had occurred, (2) understanding what trauma is and the dynamics within specific types of harms (including a subcategory focused on understanding childhood trauma and abuse, which was a profound commonality among submissions), (3) navigating relationships with family, friends, and future partners after trauma, (4) managing emotions and bodily reactions to trauma, (5) disclosing trauma to loved ones and seeking help, (6) finding and embracing healing, and (7) supporting survivors and being an ally. Two inquiry areas that did not meet the threshold for a subtheme, but still may warrant additional attention include questions pertaining to systemic responses to sexual assault and institutional betrayal. See Table 3 for example questions relating to each theme and subtheme.

**Table 3:** Example questions across themes and subthemes.

Theme	Subtheme	Example question
Validating Experiences and Recognizing Harm	“Is this normal?”	Is it normal to have experienced pleasure during an assault? I think I may have had an orgasm, but I’m not sure. I feel so betrayed by my body. I’m just too embarrassed and ashamed to ask my therapist.
	“Is this my fault?”	
	“Am I a bad person?”	Why do I keep making myself relive what happened, as well as versions of the event that did not even happen and are either infinitely worse or are completely different? I keep making myself read or create stories about it and I keep getting lost in daydreams I do not want to have. Does it make me a bad person?
	“Does this count?”	Was I still sexually assaulted if I wasn’t raped, but was touched sexually without my consent?
Understanding Trauma and Specific Types of Harm	Coercion and psychological abuse	
	Childhood trauma and abuse	What is child-on-child sexual abuse (COCSA)? What is considered COCSA between same aged, pre-adolescent children? Are there any resources or research to help determine what behavior is healthy vs. abusive?
	Relationship violence	What is the cycle of abuse and how can this cycle be broken?
	Defining trauma and abuse	When does sexual assault count as rape?
Navigating Relationships After Trauma	Building and maintaining relationships	How do I continue to heal while attempting to build a new relationship? How do I break old habits of dependency or fear of my partner?
	Re-evaluating relationships	How do I deal with family members who do not take my traumatic history seriously?
	Sex, sexuality, and intimacy	I’m a sexual assault survivor that is currently in a healthy relationship. My boyfriend is aware of what I experienced as a child. He’s timid when it comes physical touch because he does not want to trigger any past trauma. How can I reassure him that it’s okay to show affection?
	Setting boundaries	What should I do if my partner pushes my boundaries in a way that makes me uncomfortable?
Managing Emotions and Bodily Reactions	Navigating triggering situations	Every year on the anniversary of my trauma, I feel a fresh pain deep down. I’m even triggered by holiday preparations, certain drinks, and other things that remind me of the season. How can I best support myself on an anniversary I do not want to remember?
	Making sense of emotions and reactions	I was coerced into having sex with my ex-husband. I feel confused, ashamed, depressed and it’s all I’m thinking about. I have moments when I’m okay and then it hits me all of a sudden. Part of me thinks it wasn’t that bad because I said fine in the end after saying no repeatedly. Why do I feel like this? I can hardly breathe…
	Managing emotions and reactions	How can I deal with “bad days” when I am healing from trauma?
Disclosure and Seeking Help	Mobilizing Your Support System	I want my family to know the full story about the sexual assault I experienced, but I risk losing them in the process. My cousin was the one that harmed me and my family agreed not to talk about it so she will not go to jail. How do I get closure when everyone around me wants to keep it in the dark?
	Accessing professional support	I find getting after care counseling from trauma-informed counselors very difficult in my state. Do you have any advice?
	Disclosing trauma to others	How can I explain to my Mom that I mentally blocked out my sexual assault for two years?
Embracing Healing	Understanding the healing process	So how do you heal from trauma?
	Engaging in healing activities	How can I celebrate the small ways I am healing from trauma?
How to Support Survivors and be an Ally	Supporting Survivors	My girlfriend told me that before she was with me, she was sexually assaulted. How can I help her?
	Understanding Survivors	Why might survivors of gender-based violence may feel hesitant to report this human rights violation?

### Educational exhibits

A unique feature of the Our Wave platform is the opening of pathways for creating a supportive community. Our Wave actively collaborates with local activists, artists, researchers, and survivors to develop interactive educational exhibits that explore the complex intersections of survivorship, trauma, violence, and healing. Through these partnerships, Our Wave harnesses diverse perspectives and expertise to create engaging and informative experiences that foster empathy, understanding, and awareness. By integrating survivor narratives, artistic expressions, and research findings, these exhibits provide a platform for dialogue and reflection, challenging societal norms and promoting meaningful change. Through interactive elements such as multimedia installations, workshops, and community events, Our Wave strives to amplify marginalized voices, empower survivors, and catalyze collective action toward ending SV and promoting healing-centered approaches. Currently there are four exhibits on the website: a video describing four survivors unique healing journeys, an art gallery featuring art from male survivors, a “what were you wearing” exhibit with photographic images of people’s clothing that they were wearing during their sexual assault to combat victim blaming and survivor stereotypes, and a photography exhibit that shares what healing means to survivors with corresponding photographs taken by them to share their healing moments.

### Website statistics and platform user engagement

Website visitors come to the Our Wave platform through a variety of pathways, from direct searches via an internet search engine such as Google to references from local or national service agencies.

As of October 2024, 741 stories have been submitted directly to the Our Wave community, while 451 stories have been submitted to partner organizations that use the Our Wave platform to support their mission. Of these submitted stories, 86.7% of story submissions provide consent for the use of the materials in the story for further research.

Among the stories submitted to both the Our Wave community and partner organizations, a majority include additional, optional features added at the discretion of the author. 739 stories out of the 1,279 submitted (57.78%) included a “Message of Healing,” or a reflection by the survivor on what the healing process means to them for other survivors to read. Similarly, 671 stories (52.46%) included a “Message of Hope”—a direct message of support for another survivor in the online community. This greater than 50% prevalence in the use of these features demonstrates a very important aspect of the story disclosure and submission process, namely, that for those sharing their story, it is also important to express solidarity and a belief that healing is possible with a broader community.

**Figure 2:**
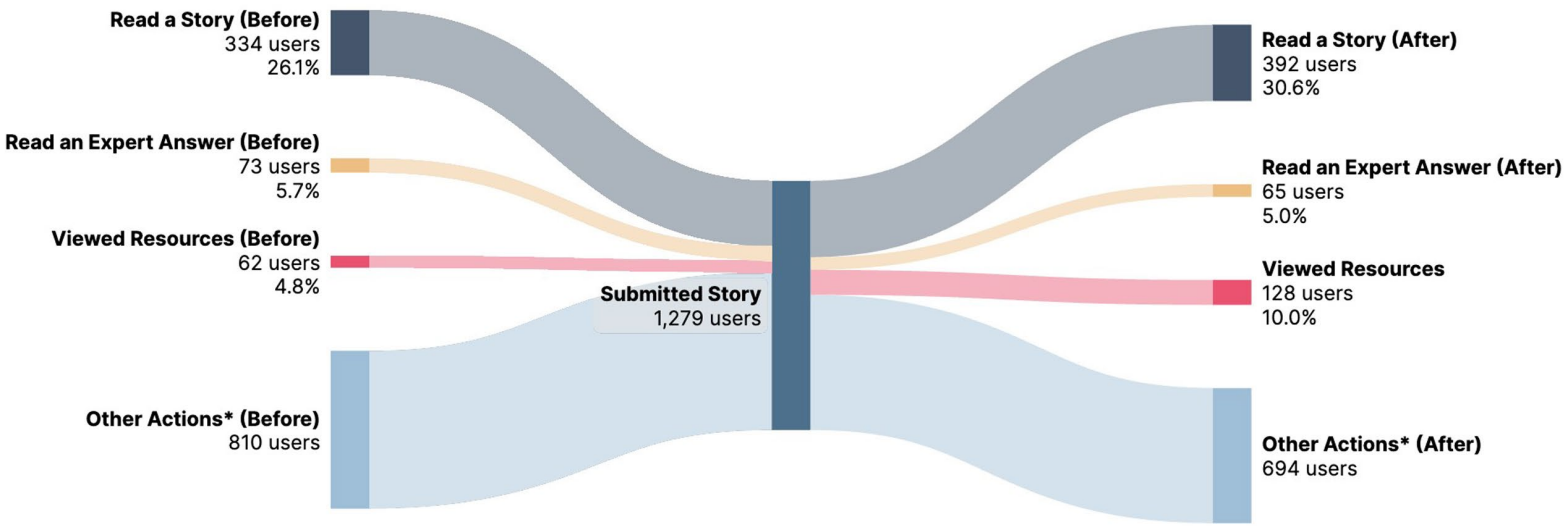
Website visitor “journey map”.

Analysis was also conducted on the overall experience of a visitor to the Our Wave platform. To do this, anonymous metrics were collected on which stories, questions, and resources a visitor read or interacted with, and whether they submitted a story to the platform. The result of these metrics is a “journey map” (Figure 2) for each visitor detailed their individual experience navigating the website and engaging with survivor content.

This “journey map” highlights an important facet of the story submission experience, namely the types of content that are inherently engaging to visitors before and after they submit their story. One-quarter (26.1% or 334 users) read at least one survivor story before submitting their own story. 30.6% of users who submitted their story went on to read at least one story after (392 users). Similarly, 5.7% of story submitters read at least one expert answer to a survivor question before submitting their own story (73 users), while 5% read an expert answer after submitting (65 users). This trend of increased engagement after the act of story submission is also reflected in views of the Our Wave resources hub. 4.8% of story submitters viewed the resources hub before submitting their own story (62 users), while more than double that (10.0% or 128 users) engaged with the resources hub after submitting.

In total, 416 total survivor stories were read by survivors before submitting their own story, while 575 total stories were read by survivors after submitting their own story. In a similar way, 111 total survivor questions and expert answers were read by survivors before submitting, and 179 total were read after. Table 4 list the number of page views, as of October 2024, for the most accessed questions on Our Wave website. Overall, the analysis in Figure 2 and Table 4 points to a strong interest by visitors that submit a story to continue engaging with the survivor-centric content on the website, and that the act of story disclosure is a motivating factor to build continual engagement for visitors.

**Table 4:** Most commonly accessed survivor questions on the website.

Question	Number of page views, as of October 2024
Why might survivors of gender-based violence may feel hesitant to report the human rights violation?	12,810
Why do I get sexually aroused when thinking about my childhood sexual assault?	8,322
Is it normal to have experienced pleasure during an assault? I think I may have had an orgasm, but I’m not sure. I feel so betrayed by my body. I’m just too embarrassed and ashamed to ask my therapist.	4,621
I think I might’ve been groomed and sexually abused as a child, but my memory feels very scrambled. I feel guilty for thinking I might’ve been assaulted, what do I do?	4,146
I was kissed by someone while I was drunk. At the moment, I did not say no and was into it. However, once the alcohol wore off, I wished it had not happened. When I confronted the person and told them they should not have engaged sexually with someone who was drunk and that the only appropriate thing to do with a drunk person is to help them walk or get somewhere safe, they responded by saying that I was the one who approached them repeatedly and did not let them leave, even tearing up when they tried to go. Does this justify them kissing a drunk person? Does it make them less at fault and me more responsible?	3,194
My girlfriend told me that before she was with me, she was sexually assaulted. How can I help her?	2,889
In cases of child-on-child sexual abuse (COCSA), is it okay/normal to continue to be friends with the other child and have it be water under the bridge?	2,888
I got assaulted, but I do not really feel anything…is something wrong with me?	2,865
Child-on-child sexual abuse (COCSA): Can a victim be older than their perpetrator?	2,803
What are ways women can protect themselves against female abuse?	2,795

### Preliminary impact assessment

After story submission, website visitors are offered an opportunity to respond to an anonymous survey for feedback about their experience engaging with the platform. Using a HotJar survey tool, three questions were included: (1) Where exactly did you first hear about us? (2) What, if anything, did you like/dislike about the website? (3) What, if anything, would you consider missing right now (features, etc.)?

Between October 2020 and November 2024, 288 survey responses were logged. No information is preserved regarding relationships between survey responses and specific stories, preserving complete anonymity. Table 5 documents the themes identified in these responses with corresponding quotes.

**Table 5:** Impact assessment themes and quotes.

Theme	Quotes
Theme: Emphasis of the value of being able to post one’s story in one’s own words	“I like the fact that I can finally be able to tell my story”
	“This is a place to open up”
	“I really liked that you allowed me to share my story without any hesitation or forms or approvals… There’s a beautiful freedom in that.”
	“It is a wonderful website and place to allow Survivors have a voice as in reality there is no justice to what happened to them only memories and the long process of Healing.”
	“I really liked that you allowed me to share my story without any hesitation or forms or approvals… There’s a beautiful freedom in that.”
	“I was looking at sexual assault stories and it made me want to share my mine”
	[What did you like about the website]: “Like the obvious… a place to share my story”
	[What did you like about the website]: “I like how it’s an open space for everyone”
	[What did you like about the website]: “it’s a good way for survivors to express what she went through”
	“I had written my story and was trying to find a positive place to post it. As I was doing my research I found ourwave… The positivity and inclusivity are a big plus. The fact that it’s anonymous is a plus!”
	[What did you like about the website]: It’s EXISTENCE! It’s wonderful for people to be able to come anonymously share with others who can relate.
	“Finally … someone somewhere to Explore and not be judged I don’t have to convince anyone about my life… seems this site has no judges and support instead of blame”
	[What did you like about the website]: “Anonymous opportunity to share stories”
	[What did you like about the website]: “telling a story”
	“I like the concept of sharing not only the story, but also the healing part”
	“I really need to breathe by telling the world, looking for input, hopefully, that will allow me to breathe”
	“My first time on here and what caught my attention is sharing my story because I was abused.”
Theme: Emphasize the value of reading posted stories	“I like how other people share their stories. It makes me feel less alone.”
	“I like how survivor’s have the opportunity to anonymously share their stories, it’s very powerful.”
	[What did you like about the website]: “survivors sharing stories, having a safe place to share and feel heard”
	[What did you like about the website]: “Only yesterday did I find this site. So far I feel better just for having found it.”
	[What did you like about the website]: “Ease of access and being able to see so many touching stories”
	“I cannot talk about it so it helps to listen to those who can and have found some peace. I like the anonymity and therefore do not think I’ll register to this site, but maybe in the future I will be able to.”
	“I like that it spoke truth about how I felt”
	“like the stories, resources, safe space”
	[What did you like about the website]: “actual experience and insights of people seeking healing”
	“I like the meaning and all stories”
Theme: Story submitter expressing care and concern for impact of the story on the readers or listeners	“Thanks I just want to tell my story, I’m afraid it could be very upsetting to anyone who hears it, no one knows I’m coming to terms with it myself But it could be a shock to people I think possibly (like an undullable mark, something that you cannot forget) I figured that a place like this would be a safe place to tell my story, and I wonder if it could possibly be of some value to another person I’m planning to share it with the perpetrator.”
	“I plan on writing my story today. I have never told my story, only parts with words I thought people could tolerate.”
Theme: Community and support for story writers	“I want to comment solidarity when I read about others”
	“I liked the post set up and the positive prompts before the stories. I wish there was more interaction opportunities between survivors”
	“Mabe it would be helpful to make searching for certain stories easier, incase someone used a “"burn”" email and want to go back to a certain story. Liked that could be story, poem or whatever shared… [would recommend] “Better way to search stories maybe date the year it was written”
	[Missing features]: “A chance it build community”
Theme: Site visitors looking for community interaction and for a way to engage with the stories	“Follow up for the victims to have help seeking legal help to pursue Justice.”
	[What do you consider missing]: “Maybe a way to highlight and save some pieces of the stories and collect them.”
	[What do you consider missing]: “Comments of some sort or ability to post threads”
	[Missing features]: “Can members connect if they wish?
Theme: Interest in stories posted by persons who are comfortable disclosing their names and/or interest in posting one’s one story under one’s real name	[Recommend for the website to include]: “Being able to have non-anonymous posts if a person is brave enough.”
	“I dislike not being able to include my first name in my submission.”
	“I like the site but would prefer that I could use my own first name in my story.”

Content analysis of the survey responses followed Clarke Braun’s (27) thematic analysis framework. Codes were generated inductively, identifying meaningful units of text related to users’ experiences with the platform. Codes were then grouped into preliminary themes, which were reviewed and refined through team discussion. This iterative process resulted in the identification of six major themes: (1) value of story posting, (2) importance of reading others’ stories, (3) concern for impact on readers, (4) desire for community support, (5) interest in community interaction, and (6) perspectives on anonymity versus identification. Each theme was supported by multiple illustrative quotes to ensure themes were grounded in the data.

The responses show that story submitters deeply value the opportunity to share their experience. As one responder wrote, “I really liked that you allowed me to share my story without any hesitation or forms or approvals… There’s a beautiful freedom in that.” Respondents emphasized that reading others’ stories serves as support and encouragement. Some visitors make the decision to share their own story after reading others’, noting “I was looking at sexual assault stories and it made me want to share mine” and “Finally…someone somewhere to explore and not be judged.” Other visitors who were not ready to disclose found value in reading stories, with one sharing “I cannot talk about it so it helps to listen to those who can and have found some peace.”

Survey responses also emphasized interest in community interaction, with feedback requesting more opportunities for survivors to connect. While most users valued anonymity, some expressed interest in non-anonymous posting options. While Our Wave currently remains fully anonymous, future development in coordination with community partners may consider including options for non-anonymous story sharing.

### Ethical considerations

The design of the Our Wave platform adheres to trauma-informed principles, prioritizing survivor safety, dignity, and autonomy. All story submissions are anonymized by redacting personally identifiable information, and sensitive data is securely stored using industry standard encryption protocols. Regular security audits and restricted access to data ensure confidentiality. Participants are informed about how their data will be used for research, with an option to consent to research purposes. Due to the anonymous nature of the platform, traditional signed Informed Consent and Institutional Review Board review do not apply, but ethical guidelines from the U.S. Department of Health and Human Services (18) guide our practices.

The platform’s data security measures include encrypted data exchanges and secure login protocols to prevent breaches. Survivors retain control over their content, allowing them to edit, update, or delete their stories, ensuring ongoing autonomy and privacy.

Aligned with community-based participatory research (CBPR) principles, the platform empowers survivors by enabling control over how their stories are shared. As the platform grows, future efforts will include partnerships with survivor-led organizations and Community Advisory Boards to further ensure the needs and wellbeing of survivors are prioritized. Our Wave is committed to safeguarding survivor confidentiality, ensuring robust data security, and creating a supportive space that honors survivors’ voices.

## Discussion

The Our Wave platform exemplifies how technology can be leveraged to support survivors of SV in their healing journeys. By providing a safe, anonymous space for survivors to share stories, ask questions, and access resources, Our Wave addresses critical gaps in traditional support systems. This case study extends prior research on online trauma disclosures, such as those on social media and platforms like Reddit (16, 19, 20), which highlight both opportunities and limitations for survivors. While online platforms offer advantages such as visibility management, connectivity, and asynchronicity, they also pose risks, including the lack of non-verbal cues and the potential for retraumatization and irreversible disclosures (20).

Our Wave addresses these concerns by maintaining complete anonymity for users’ stories, reducing risks associated with social stigma and retraumatization. Additionally, the platform provides survivors with control over their narratives, allowing them to edit, update, or delete their stories at any time. This autonomy helps mitigate the concern of irreversible public disclosure (20), offering survivors a sense of agency over their stories. Users also have the option to share their experiences privately via email or publicly through social media, offering flexibility in how their disclosures are managed and shared.

The platform’s focus on anonymity and user control is reinforced by the findings from our exit survey, which showed a strong preference for these features among respondents. Although the anonymized nature of the survey limits the depth of our understanding, these results suggest that survivors are particularly concerned with privacy and the ability to control future disclosures. Furthermore, the moderated, trauma-informed environment on Our Wave helps ensure that survivors are met with empathy, reducing the risk of negative reactions or “trolling” that can exacerbate PTSD symptoms (8). This careful moderation distinguishes Our Wave from more general social media platforms, where hostile responses can further harm survivors.

Engagement with the platform, demonstrated by the over 1,200 shared stories and 100,000 community members, emphasizes the growing need for survivor-centered online spaces. The anonymous questions submitted by survivors reveal diverse concerns and challenges that align with research on the long-term impacts of trauma (4, 5), while also highlighting issues that are difficult to address outside of anonymous spaces. The platform’s ability to tailor resources to these specific needs underscores its unique role in bridging gaps in existing support systems.

While Our Wave has made significant strides in creating a supportive and trauma-informed space, it is important to acknowledge the limitations and areas for future development. Accessibility remains a key issue, particularly for survivors who may face barriers related to technology access, disability accommodations, or language. As the platform continues to grow, efforts to ensure broader inclusivity, including representation across diverse racial, ethnic, gender, and sexual orientation groups, will be essential. Research also suggests that marginalized communities face compounded barriers to accessing support (4), and ensuring the platform meets their unique needs will be vital to expanding its impact. Additionally, the platform’s ability to verify the effectiveness of the resources and referrals provided will need to be enhanced as it scales, ensuring that survivors are connected to the most appropriate and effective services.

### Practical implications

The findings of this case study offer valuable insights for the future of digital interventions aimed at supporting SV survivors. As a survivor-centered platform, Our Wave prioritizes anonymity and confidentiality, which are crucial for creating a safe and empowering space for survivors to share their experiences. Moving forward, efforts should focus on scaling the platform to reach a broader audience, ensuring it remains accessible across various devices and to users with different technological capabilities. Adding language options and accommodating people with disabilities will be key to making the platform more inclusive.

The platform’s user-selected tags feature, which allows survivors to articulate facets of their identity and healing journey, could be expanded to allow for even more nuanced self-expression. In addition, integrating peer support features—such as real-time chat or survivor groups—could strengthen the sense of community and foster shared healing experiences among users. These features would further enhance the platform’s role in providing not only individual support but also collective empowerment.

A crucial next step for the platform is the development of referral systems that connect users with professional support services. This would ensure that survivors are not only supported through the platform’s self-help features but also have access to expert care when necessary. Further, Our Wave’s approach to collaborating with activists, artists, and researchers provides a model for expanding partnerships and co-creating survivor-informed resources. This collaborative approach should continue to grow as the platform evolves to meet the diverse needs of SV survivors.

Future research is needed to explore how digital platforms like Our Wave can better engage marginalized communities, including people of color, LGBTQIA+ individuals, and people with disabilities. These groups often face unique challenges in accessing support, and it is vital to design features that are culturally and contextually relevant to their needs.

### Future applications

The findings of this study have broader implications for public policy, clinical practice, and SV prevention programs. In terms of public policy, this study highlights the importance of data protection laws that ensure the confidentiality and safety of survivors in digital spaces. As more survivors turn to online platforms for support, it is essential that policy frameworks safeguard their anonymity and provide survivors with control over their personal information.

In clinical practice, the insights gained from platforms like Our Wave can inform trauma-informed care approaches. Mental health professionals can leverage platform data to better understand survivor concerns and adapt their therapeutic practices accordingly. The platform’s anonymous questions and responses could inform clinical interventions by highlighting the specific support or areas of education survivors are seeking in their healing process.

For SV prevention, the educational exhibits on Our Wave provide a model for raising awareness and addressing victim-blaming narratives. These exhibits could be integrated into public health campaigns to promote empathy, understanding, and social change. The combination of survivor stories, artistic expressions, and research findings presents a powerful tool for shifting societal attitudes toward survivors of SV.

Looking forward, Our Wave plans to expand its impact by enhancing accessibility through mobile and multilingual features, enabling it to support an international user base. Future iterations of the platform will also integrate real-time consultations with experts, webinars, and additional educational resources to further assist survivors in their healing journeys.

Further research will focus on evaluating the long-term impact of the platform on survivors’ mental health, social support, and engagement with formal services. Key areas for exploration include identifying early intervention opportunities through the analysis of survivor narratives and comparing the effectiveness of Our Wave with other online support platforms. Future research should also explore best practices for content moderation and trauma-informed online support to help establish standards for similar platforms.

As part of its commitment to holistic survivor support, future directions also include integrating intervention features to assist survivors in crisis. Linking users to mental health services, legal aid, or emergency counseling could provide more immediate support to those in need. Collaborations with public health institutions and advocacy organizations will be crucial in expanding the platform’s impact and integrating it into broader systemic interventions for survivors of SV.

## Conclusions: the challenges and opportunities for supporting survivors online

The Our Wave platform represents an innovative approach to survivor support and advocacy, with the potential to drive meaningful change in the lives of survivors of SV. Through its features—such as anonymous story-sharing, survivor Q&A, and collaborative educational exhibits—Our Wave offers a safe, inclusive, and empowering space for survivors to share their experiences, access resources, and build community.

The platform’s growing user base and the positive impact it has made thus far underscore its ability to meet the diverse needs of survivors and contribute to a broader culture of healing and resilience. With sustained investment and support, Our Wave is well-positioned to expand its reach and deepen its impact, creating opportunities for systemic change in the way SV is addressed. By continuing to promote survivor-centered approaches to healing and fostering greater collaboration among communities, Our Wave has the potential to reshape the landscape of SV support, offering survivors a powerful tool for recovery and empowerment.

## Data Availability

The original contributions presented in the study are included in the article/supplementary material, further inquiries can be directed to the corresponding author.
